# A Clinical Lecture on Opthalmology

**Published:** 1881-11

**Authors:** D. B. St. John Roosa


### Independent Practitioner.

VOL. II.——*November*, 1881.——NO. 11.

ARTICLE I.

### A CLINICAL LECTURE ON OPHTHALMOLOGY.

### INJURY TO THE EYE.

BY PROF. D. B. ST. JOHN ROOSA, M. D.

Delivered before the Medical Students of the University of the City of NewYork, November 2, 1881.

This man comes here from the Manhattan Eye and Ear Hospital. I have sent him here to-day for two purposes. In the first place that I may learn how he has gotten on since treatment which was prescribed for him. In the second place that you may study the case with me and learn, if possible, what is the best method, on the whole, for treating such affections.

Although I have often told you that the story of a patient affected with a disease of the eye, is that one affected with a disease that you can examine yourself with your own eyes.

The reason that the patient's history becomes so important in pthisis, in affections of the chest in general, in syphilis, etc., is that we cannot entirely rely on our objective examinations for our proofs of the disease. Now, with diseases of the eye, there is so much evidence that we care very little for what the patient says—although there is always a certain value to be attached to his statements.

I remember an old gentleman came to my office one day, complaining of his eyes, and I tested his vision. I immediately learned that his sight was very much affected. But before I examined him with the opthalmoscope, I allowed him to tell his story. And there was nothing of importance, gentlemen, in the account of his own symptoms. He told us how the light had pained his eyes—about his gradual failure of sight, of his perception of colors, and so forth. It seemed very interesting to him. But we found an opacity of the lens of each eye when we examined him. He had cataract.

So all of that history was of no account whatever. The only thing of value was the amount of opacity in the lenses.

Thus we have the advantage in diseases of the eye. In many accidents to or diseases of the eye, we are not dependent upon the accounts of the patients themselves.

This man is a blacksmith by occupation, and thirty-one years of age.

He has given a clear account of his injury, but there is a technicality in it that we don't all understand. He says that he was welding two bits of iron together, striking while the iron was hot, striking quick and fast, and sand sparks came up and hit his eye.

I don't know what they are. Will you explain (addressing student)?

"Sand that is put on for making the iron weld."

So that was the kind of foreign body that hit his eye.

According to this man's statement this was a case in which this foreign body hit his eye—it burned his eye.

Now let us see what part of the eye-ball was affected.

Dr. V. here (who has been attending him) says that he is so much better to-day that he is not a good case to show.

Now" (addressing patient) "just shut your eyes quietly." You see there is a little more blood in the left eye than there is in the right.

Then please write down "eyelids of left eye a little red."

The ordinary facts of nature are not always well studied. Just think for a moment what gives you the natural pinkish hue to the skin. It is the blood-vessels. These are more numerous in the left eye than in the right. We find, in the next place, secretions between the lids, and muco-pus on the lids. I will wipe all that off, and will open the lids. When I open the lids in that way and look at the ocular conjunctiva, what am I thinking of? I have in my mind a picture of a healthy ocular conjunctiva.

I ought to know how a healthy one looks, if I am to treat a diseased one. Suppose that I only had received a theoretical education. I would know the anatomy of a healthy conjunctiva, at least. Suppose I want to know whether there is anything wrong in the condition of this ocular conjunctiva, (looking at student's eye). I compare its appearance in my mind with that of a healthy conjunctiva.

A healthy conjunctiva is white, transparent, so much so that the white sclerotic shows through it in every one. If I look close I can find that I can distinguish individual blood-vessels—not many of them. A beautiful tracing. Much like the many graceful branches of a tree. But the general color is white, but in this man the general color is red. And so we will say that the color of this conjunctiva is red. It is so red that when I look closely in the patches I cannot see the individual blood-vessels.

Now I will look at his cornea to see what I can see there. I will bring into play a better means of observation. I am going to concentrate the light of the candle with a two-inch burning-glass or lens.

How does a healthy cornea look?

I remember that it is perfectly transparent.

Through it I see what?

I see a black hole called a pupil, and I see a beautiful membrane called the iris. An iris is so named after the rainbow, because it has different colors in it. It might much better be called a diaphragm, regulating the amount of light that enters the eye.

Now we will bring the lamp for this direct illumination. You will see how the doctor holds it. You will see this method, which you will find extremely valuable. Everybody can have an artificial light. Every physician should carry a magnifying glass. Now we have a more concentrated light on the cornea. It does not hurt the eyes. It does no harm. You need never be afraid of doing harm in that way. (Addressing patient.) "Now look up if you please, now look over toward the lamp. Dr. V. tells me that yesterday the scar of the burn was directly across the cornea. He says that the man had an opacity across the cornea one-eighth of an inch wide. He saw it about an hour after it had happened. He found that the epithelium of the cornea had been burned off by this sand. You know the tissues of the cornea. First the corneal epithelium, then the anterior elastic lamina, then the proper membrane of the cornea, and lastly the membrane of Descemet. Dr. V. saw that this layer of epithelium had been burned off.

There was quite a scar there, just as if a man had taken a piece of nitrate of silver and touched it over with it.

I still see, although his eye is so much better than yesterday, that the epithelium is a little roughened, and I judge that we still have disturbance in the cornea. And you see there are increased secretions through the whole conjunctival sack.

Wipe some of the secretions away. I find some dirt or some foreign substance. We find a piece of coal or steel has gotten in there.

I find the pupil has been dilated. This is from atropia applied yesterday. Now suppose that that man had come into my office and he had been my patient? What would have been my diagnosis? It would have been plain.

This man has had an injury to the eye from a bit of hot sand or metal, which has produced a wound of the cornea, and which has also produced inflammation of the conjunctiva. A wound of the cornea which has produced keratitis and conjunctivitis, manifested by redness and increased secretion. Now I have heard nothing from this man except how he received his injury. He could tell me a very interesting account of how many hours he laid awake last night from pain and so forth. The objective symptoms are however all that are important to you, and you wish to know how his eye may be cured. That is the object of all medicine. The object of all medicine is to make a man better. To cure, alleviate or eradicate his disease in order to get him well.

Now I want you to tell me what you would do for this man. Then I will tell you what was done.

Let us see what we would do (addressing student): "Now Doctor, what would you do?" "I would apply a dressing of cold water."

"And how would you have him bathe his eye with cold water?" Ans. "By throwing some against his eye with his hand, or by applying cloths dipped in water. I would do that for the first few hours after the injury."

"Now Doctor, what would you do, suppose that you had used the cold water ; what other thing would you think of doing?"

(Addressing patient). " This eye hurts you some, doesn't it?" Ans. " Yes, sir."

" Did it hurt you very much yesterday ?" " Yes, sir."

Now in the light of that inquiry, what would you do besides applying cold water ? Now if you are not familiar with the ophthalmic therapeutics, I will tell you what any ophthalmologist would do to mitigate pain. He would think of the drug, sulphate of atropia, and would generally apply it for the relief of pain, about two grains to the ounce, for experience shows that that is usually sufficient to relieve pain. 1-20,000 of a grain would do very well to dilate the pupil.

Here are two things : In the first place cold applications ; in the second place sulphate of atropia. What other treatment would you undertake ?

I will make another inquiry to see whether anything else would suggest itself.

(Addressing patient.) " My friend, did you go back to the shop to work ?" " No, sir, I did not work ; I went back there to tell them how I had been treated, and to tell them the extent of the injury.

" What did you do next ?" Ans. " After that I went home and bathed it in cold water and used a drop of atropia in it."

" What is the next thing I would advise for him ?" Ans. " Rest."

Yes, that is very important. Suppose that he had gone right back to the fire and commenced blowing the bellows or hammering over an anvil; the heat that was borne by the healthy eye would not be tolerated by the diseased one.

Here the indications are to keep his eye away from such things. Besides rest, what else can we do ? We can shield it from light with a shade.

Now let us ask him how he is getting on.

If you inflict an injury upon a cornea, it is, to a certain extent, irreparable, as a blow upon transparent glass.

You may mitigate the damage, but you cannot always wholly repair it.

He came in there yesterday suffering very much indeed, and he had a great scar over his cornea. All the nerves of his cornea were responding to his injury.

(Addressing patient.) " Did you feel any better after what was done for you yesterday?" Ans. " Yesterday afternoon it hurt just about as much as it did the first few minutes after the injury. It kept me awake the greater part of the night."

That illustrates what I want to teach you. To-day he is a great deal better. His cornea is so clear to-day that it is difficult to see where there has been an injury. There is one spot of little account to show that there has been any injury, and that is right on the edge.

The object of all our treatment is to save his eye this time for him. He can't afford to lose his eye. A day of his time would represent a week or two weeks of your time taken away.

We must endeaver to get his eye in a condition that he can work with it as soon as possible. I wish I knew why the sulphate of atropia is an anodyne for the eye. It contracts the blood vessels of the eye is one reason given. It relaxes the ciliary muscle, it stops its action.

It puts his eye in a sling, as I am in the habit of saying. His eye has been put at rest.

Now I do not recommend him to use cold water to-day, as the stage of increased temperature has passed away and the stage for cold has passed away. Let him now use warm water. Let him keep out of the dust. I think that he will be at work again without any more trouble with his eye than if this injury had not been. Fortunately it was only through the epithelium, and nature quickly produces an epithelium, if you have not interfered so as to prevent her doing her beneficial work.

What did we refrain from doing? We did not put on poultices on this eye. We did not put on bread and milk. We didn't put on oysters. We could have given him a night's rest if you had put on oysters or poultices of any kind, but he would have slept while his eye was being destroyed. I hope that I can show him to you next

Wednesday, if he can eat and drink well in the meanwhile. If he can be properly nourished, I hope to be able to show him to you in a week, able to do his work. The cornea will not heal unless nutrition is going on properly.

---

### TREATMENT OF OZÆNA.

In several cases of chronic inflammation of the nasal and pharyngeal cavities, giving rise to offensive discharge, Dr. Poore has found decided benefit result from the use of a stimulant and antiseptic snuff having the following formula : biborate of soda, nitrate of bismuth, of each one drachm ; disulphate of quinine, ten grains ; iodoform, five grains. This snuff has the effect of stopping the fetor and greatly diminishing the amount of discharge from the nostrlis. It is liable, as are all snuffs when used for similar conditions, to cake in the nostrils, and it is therefore necessary to thoroughly wash out the nostrils once a day. This may be done by means of a nasal douche, or the patient may easily be taught to snuff a lotion up the nose and allow it to run out of the mouth. A teaspoonful of glycerole of borax dissolved in a wineglass of tepid water forms an excellent wash for the nose, and with a little instruction patients learn how to wash out their nasal and pharyngeal cavities without the aid either of syringe or douche apparatus. In cases where the ozæna is of a simple kind, not due to caries or necrosis of bone, but rather to a sluggish, inflammatory action occurring in a scrofulous subject, considerable benefit is often derived from the administration of the sulphide of calcium in doses of half a grain (in pill), taken three times a day. It is often necessary to cleanse the nasal and pharyngeal cavities with a brush inserted through the anterior nares, and also behind the soft palate,so as to reach the summit of the pharynx. The brush may be moistened with glycerole, of tannin, and after the cavities have been cleansed a little dry iodoform may be passed into the cavities on the tip of the brush.—*London Lancet.*

